# Assessing van der Waals Corrections in the Description of Water Adsorption and Diffusion on Graphene and Hexagonal Boron Nitride

**DOI:** 10.1021/acsomega.6c02762

**Published:** 2026-06-12

**Authors:** Tulio Gnoatto Grison, Douglas Duarte de Vargas, Celso R. Caldeira Rêgo, Alexandre Cavalheiro Dias, Mateus H. Köhler, Diego Guedes-Sobrinho, Maurício Jeomar Piotrowski

**Affiliations:** † Department of Physics, 37902 Federal University of Pelotas, PO Box 354, Pelotas, RS 96010-900, Brazil; ‡ Department of Physics, 28118 Federal University of Santa Maria, Santa Maria, RS 97105-900, Brazil; § 150232 Karlsruhe Institute of Technology (KIT), Institute of Nanotechnology, Eggenstein-Leopoldshafen 76344, Germany; ∥ Institute of Physics and International Center of Physics, 564113 University of Brasília, Brasília, DF 70919-970, Brazil; ⊥ Quantum Chemistry and Thermodynamic Materials Group, Department of Chemistry, 28122 Federal University of Paraná, Curitiba, PR 81531-980, Brazil

## Abstract

A comprehensive first-principles investigation was conducted to assess the performance of distinct van der Waals (vdW) correction schemes in describing the structural, energetic, and dynamical properties of water adsorption on graphene and hexagonal boron nitride monolayers. The PBE functional was complemented by both empirical (D2, D3, D3BJ) and nonlocal (DF1, DF1C09, DF2, DF2C09) functionals to assess how dispersion affects adsorption and diffusive behavior at solid–liquid interfaces. The combination of static structural optimizations, *ab initio* molecular dynamics, and climbing-image nudged elastic band calculations provides a comprehensive picture of the weak physisorption profile. The analysis reveals that even though empirical schemes enhance binding relative to bare PBE and generally reproduce the correct energetic scale, their accuracy varies depending on the substrate, whereas selected nonlocal vdW-DF approaches reproduce equilibrium geometries and diffusion trends reasonably well, albeit with larger deviations in adsorption energetics relative to high-level many-body benchmarks. Among the tested schemes, D3 and D3BJ provide the closest agreement with benchmark adsorption energies derived from diffusion Monte Carlo and random-phase approximation calculations. DF2 reproduces the weak physisorption regime and equilibrium adsorption geometries with reasonable accuracy; however, it systematically predicts stronger interaction energies than the many-body reference methods and slightly amplifies the energetic distinction between graphene and hBN. *Ab initio* molecular dynamics simulations confirm the thermal stability and high lateral mobility of water on both surfaces, which are associated with shallow diffusion barriers below 20 meV. These findings highlight the critical role of long-range correlation in modeling polar molecules on 2D materials and establish a quantitative framework for selecting vdW corrections in density functional theory studies of solid–liquid interfaces.

## Graphical abstract

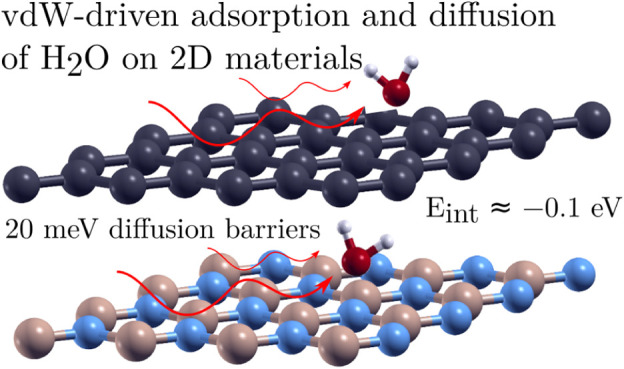

## 1 Introduction

The intermolecular interactions underpin a wide range of physical and chemical phenomena, ranging from molecular cohesion in condensed phases to adhesion and transport at solid–liquid interfaces.^1,2^ Among these interactions, van der Waals (vdW) forces play an important role. Originating from correlated charge-density fluctuations, they are long-ranged and weak in energy magnitude when compared, for instance, to covalent or ionic bondings.^3,4^ Despite their subtle energetic scale, vdW interactions can, in some cases, govern adsorption, cohesion, and diffusion processes in molecular and condensed-matter systems, which are crucial for the description of catalytic properties, such as efficiency, selectivity, and mechanistic description at the atomistic level.^5,6^ However, the development of computationally efficient first-principles approaches, as well as the correlation between theoretical background and the performance of vdW methods implemented in several numerical codes, depends on understanding how different vdW methods operate in specific physisorption processes and their impact on atomistic properties.

Two-dimensional (2D) materials provide a stringent platform for assessing the accuracy of vdW treatments. In particular, graphene (Gr) and hexagonal boron nitride (hBN) are representative examples of atomically thin layered systems, combining some interesting features, e.g., atomically flat surfaces, high chemical and mechanical stability, and different electronic characters (semimetallic for Gr and wide-gap insulating for hBN).^7–10^ These features make them well-suited to isolate the dispersion-driven effects in weak adsorption regimes. Consequently, water adsorption on these substrates is not only a fundamental scientific problem but also central to applications in nanofluidics, electrochemistry, catalysis, and environmental sensing, where interfacial structure and molecular mobility play a significant role.^2,11,12^ However, the accurate theoretical description of weakly bound water–surface systems remains a persistent challenge. Even though Density Functional Theory (DFT) has long been established as the primary quantum mechanical approach for modeling atomistic materials,^13–15^ with its generalized gradient approximation (GGA) functionals, such as PBE, accurately describing short-range exchange and correlation, these functionals fail to capture long-range dispersion effects.^16–19^ To overcome this deficiency, several approaches have been proposed through the inclusion of semiempirical atom-pair corrections, such as the notable Grimme’s D2, D3, and D3BJ schemes,^20,21^ as well as fully nonlocal density functionals of the vdW-DF family, such as DF1, DF2, and their C09 exchange variants.^22,23^

Although both strategies aim to incorporate dispersion interactions absent in semilocal DFT-functionals, they differ substantially in their physical formulation. Empirical schemes introduce dispersion as an *a posteriori* correction to the Kohn–Sham total energy, whereas nonlocal functionals embed long-range correlation directly within the exchange-correlation (kernel) functional. While extensive benchmarks exist, their relative accuracy and transferability across different adsorption systems and material classes remain open questions. In this context, the water-surface interface represents a particularly demanding benchmark for dispersion-inclusive methods.^24,25^ High-level reference calculations based on diffusion Monte Carlo (DMC), coupled-cluster [CCSD­(T)], and random-phase approximation (RPA) approaches consistently report adsorption energies on the order of −0.10 eV for H_2_O@Gr and −0.11 eV for H_2_O@hBN, with equilibrium oxygen-surface distances in the range of 3.1–3.3 Å.^25,26^ Previous theoretical studies indicate that the accuracy of dispersion-inclusive methods strongly depends on the specific exchange-correlation treatment and adsorption system, with both empirical and nonlocal approaches exhibiting distinct tendencies toward overbinding or underbinding.^4,6^

A substantial body of work has established the importance of dispersion interactions in describing water adsorption on graphene and related 2D materials, including systematic comparisons between semilocal, empirically corrected, and nonlocal vdW approaches. Foundational contributions based on the vdW-DF formalism,^22,23,27^ as well as subsequent refinements of the exchange component,^28,29^ have significantly improved the description of dispersion interactions in extended systems. In parallel, benchmark studies based on many-body approaches, including DMC and RPA calculations,^25,26^ have established reliable reference data for water adsorption on graphene and related materials.

Nevertheless, direct experimental measurements of adsorption energies for isolated water molecules on ideal Gr and hBN surfaces remain challenging. Experimental and combined experimental–theoretical studies consistently indicate weak physisorption, high mobility, and shallow diffusion barriers at graphene-based interfaces.^2,11,12^ Despite these advances, most previous studies have focused either on adsorption energetics or on specific methodological aspects. Systematic comparisons that span multiple vdW schemes within a unified computational framework, while simultaneously addressing structural, energetic, and dynamical properties, remain comparatively scarce.

Here, we present a comprehensive investigation of water adsorption and diffusion on Gr and hBN monolayers (H_2_O@Gr and H_2_O@hBN, respectively). By combining static DFT calculations with *ab initio* molecular dynamics (AIMD) simulations and climbing-image nudged elastic band (CI-NEB) analyses, we assess not only adsorption energetics and preferred binding configurations, but also temperature-driven diffusion pathways and barriers. The present study focuses on the dilute adsorption limit, where a single water molecule interacts with the substrate. This regime enables a direct comparison with benchmark many-body calculations and isolates the intrinsic molecule–surface interaction, providing a fundamental reference for the underlying potential-energy surface. Coverage effects and intermolecular interactions, which may become relevant at higher concentrations, are beyond the scope of this work and will be addressed in future studies. Within this framework, our results provide microscopic insight into how different treatments of dispersion interactions modulate interfacial structure and molecular mobility on 2D materials.

## 2 Methodology and Computational Details

All calculations were performed within the framework of spin-unpolarized DFT^30,31^ using the plane-wave pseudopotential method as implemented in the Quantum ESPRESSO package. The exchange-correlation energy was described by the PBE functional,^32,33^ which was combined with distinct vdW schemes: empirical Grimme’s D2 correction,^34^ D3 correction,^20^ and D3BJ correction;^21^ as well as the nonlocal density functionals vdW-DF1,^22^ vdW-DF1-C09,^35^ vdW-DF2,^36^ and vdW-DF2-C09.^23^

Ultrasoft pseudopotentials were employed for all atomic species, and a kinetic energy cutoff of 80 Ry was adopted for the plane-wave expansion, ensuring convergence of the total energy within 0.1% (1.0 meV per atom). The Brillouin zone sampling was conducted using a Monkhorst–Pack **k**-point mesh of 12 × 12 × 1 for the Gr and hBN primitive unit cells. Periodic slab models were separated by a vacuum spacing of at least 15 Å along the surface-normal direction in order to prevent spurious interactions between periodic images. Within this framework, all the structures were fully relaxed until the residual Hellmann–Feynman forces on each atom were smaller than 1 × 10^–3^ Ry a_0_^–1^. Convergence tests for the kinetic energy cutoff and **k**-point sampling were performed, with representative tests being provided for Gr in the Supporting Information (Figures S1 and S2).

The adsorption/interaction energy (*E*_int_) between the water molecule and the substrate (Gr or hBN) was defined as $E_{\mathrm{int}}=E_{H_{2}\mathrm{O@substrate}}-E_{\mathrm{substrate}}-E_{H_{2}O}$ , where negative values correspond to exothermic adsorption. Furthermore, finite-temperature dynamics were investigated using AIMD simulations in the canonical (NVT) ensemble, with temperature control via a Nosé–Hoover thermostat set to 300 K. In this protocol, each trajectory was propagated for 10 ps with a time step of 1 fs, and the diffusion coefficients were extracted from the long-time behavior of the mean-squared displacement (MSD) using the Einstein relation. Additionally, energy barriers associated with surface diffusion were determined using the CI-NEB method, connecting adjacent low-energy adsorption sites.

## 3 Results and Discussion

### 3.1 Pristine Systems

Before addressing the water interaction with the 2D substrates, a careful characterization of the pristine constituents was performed for each vdW correction here considered, more specifically, focusing on Gr, hBN, and the isolated water molecule. This step is essential to ensure internal consistency, as the equilibrium geometries and reference energies of the individual components directly affect adsorption energetics and interfacial properties. The construction of the Gr and hBN substrates follows a supercell approach designed to accommodate a single water molecule by suppressing spurious interactions between periodic images. However, the reliability of this approach critically depends on the accurate determination of the equilibrium lattice parameters, which were obtained at the unit cell level for each vdW scheme. Figures S3 and S4 in the Supporting Information display the total energy as a function of the in-plane lattice parameter for Gr and hBN, respectively. In each case, the equilibrium geometry was identified by fitting polynomial curves to the energy-volume curves.

For Gr, all vdW corrections reproduce the experimental lattice constant, i.e., *a*_0_ = 2.462 Å,^37^ with deviations smaller than 0.5%, highlighting the PBE functional robustness in describing strongly covalent π-bonded systems. Among the tested approaches, the D3BJ correction yields the closest agreement with experimental values, whereas the nonlocal DF1 and DF2 functionals slightly expand the lattice, reflecting the softer exchange component present in these formulations. For hBN, the equilibrium lattice constants, calculated across all vdW schemes, remain within 0.5% of the experimental value, i.e., *a*_0_ = 2.503 Å.^37^ Relevant to note that the DF1C09 and DF2C09 variants provide the most accurate description, which highlights the importance of exchange optimization in layered insulating systems. All the equilibrium lattice constants can be accessed in Table S1 in Supporting Information.

Once the equilibrium geometries were established, the electronic structures of pristine Gr and hBN were computed using configurations optimized with each vdW correction method. Figure 1 depicts the band structures for Gr and hBN through the PBE, D3BJ, and DF2C09 protocols, whereas the results for all vdW corrections are depicted in Figures S5 and S6 of the Supporting Information, while the band gap values for hBN are summarized in Table S2. As expected, all the vdW models keep the pristine symmetry from the hexagonal Gr structure, exerting a negligible influence on the characteristic Dirac cone for Gr and confirming that long-range correlation effects do not perturb its semimetallic π-band topology. In contrast, for hBN, nonlocal vdW functionals induce a moderate reduction of the band gap compared to PBE and empirical corrections. While PBE and its semiempirical vdW variants predict an indirect K−Γ gap of approximately 4.4 eV, the DF-based approaches reduce this value to the range of 3.1 to 3.9 eV. This reduction mainly reflects differences in the exchange component and equilibrium geometry introduced by the vdW-DF formulations, rather than a direct modification of electronic screening, and is consistent with previous hybrid-DFT and GW benchmarks reported in the literature.^38,39^

**Figure 1:**
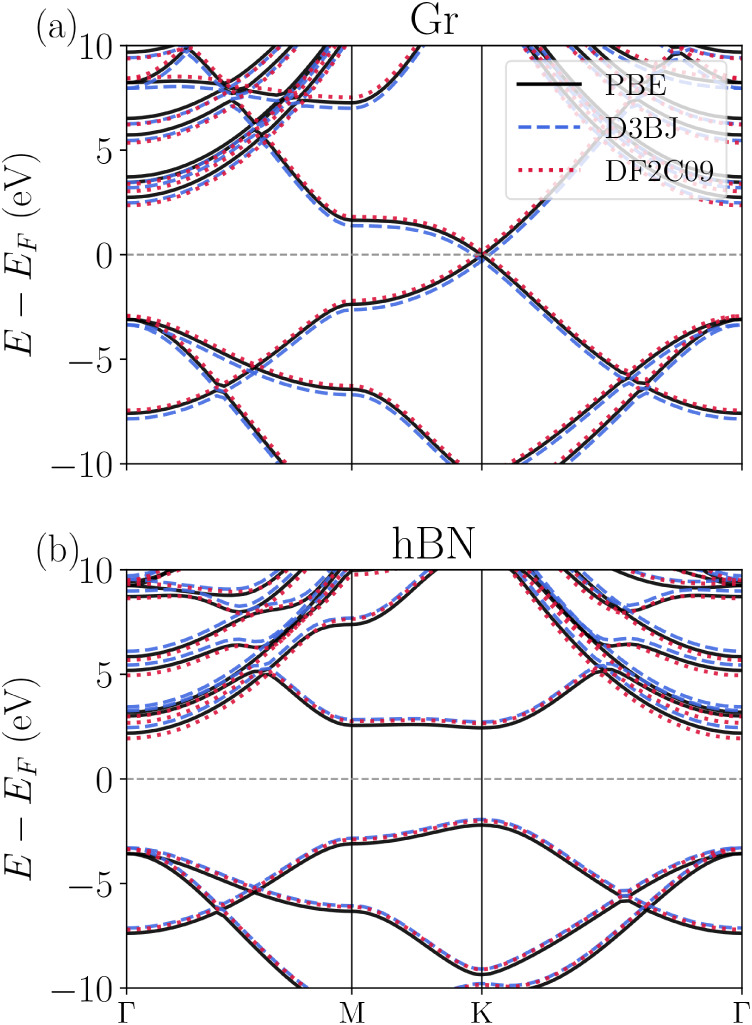
Band structure for the pristine Gr and hBN based on the optimized systems through PBE, D3BJ, and DF2C09 protocols. The Γ → M → K → Γ as **k**-path has been used to all systems.

Following the unit cell characterization, the optimized Gr and hBN lattices were replicated using a supercell approach to host a single water molecule on the surface, thus ensuring sufficient separation between periodic images. Convergence tests with respect to the lateral supercell size are shown in Figure S7 from Supporting Information, where both the total energy and the orientation angle of the water molecule are monitored as functions of the intermolecular separation. These tests demonstrate that a 4 × 4 GR supercell (32 C atoms) and a 4 × 4 hBN supercell (16 B and 16 N atoms) are sufficient to suppress artificial water–water interactions, providing a reliable platform for adsorption studies.

The structural stability of the pristine substrates within each vdW scheme was assessed through cohesive energy calculations, which follows the expected trend, i.e., empirical vdW corrections modestly enhance binding relative to bare PBE by approximately 0.05 eV per atom, whereas the nonlocal DF1 and DF2 functionals yield slightly weaker cohesion due to the inclusion of long-range correlation effects (due to differences in the exchange functional combined with the nonlocal correlation term). This behavior reflects trends previously reported for other layered materials with π-bonds, reinforcing the physical consistency of the results presented.^7^

Finally, the isolated water molecule in the gas phase was analyzed as an essential reference system. The optimized O–H bond length, HOH angle, and binding energy for each vdW correction are summarized in Table S3 of the Supporting Information. The structural parameters remain virtually unchanged across all methods, with *d*_OH_ ≈ 0.97 Å and ∠HOH ≈ 104.4°, confirming that dispersion corrections have a negligible impact on intramolecular covalent bonding. In contrast, small variations are observed in the molecular binding energy, with DF1 and DF2 showing slightly reduced magnitudes than those obtained with empirical and/or PBE schemes. This reduction is caused by the nonlocal correlation contribution, which primarily affects long-range interactions and has minimal influence on localized molecular bonds.

### 3.2 Adsorbed Systems

All adsorption results discussed in this section correspond to the dilute limit, where a single water molecule interacts with the substrate, allowing for a direct comparison with benchmark theoretical data. For the adsorption of a single water molecule on Gr and hBN monolayers, we investigated the relevant high-symmetry adsorption sites in order to assess the influence of distinct vdW correction schemes on equilibrium geometries, as well as their interaction energies and electronic redistribution. Adsorption geometries and interaction energies are summarized in Table 1, while representative optimized configurations are shown in Figure 2 (and Figure S8 and Tables S4–S7 of the Supporting Information).

**Figure 2:**
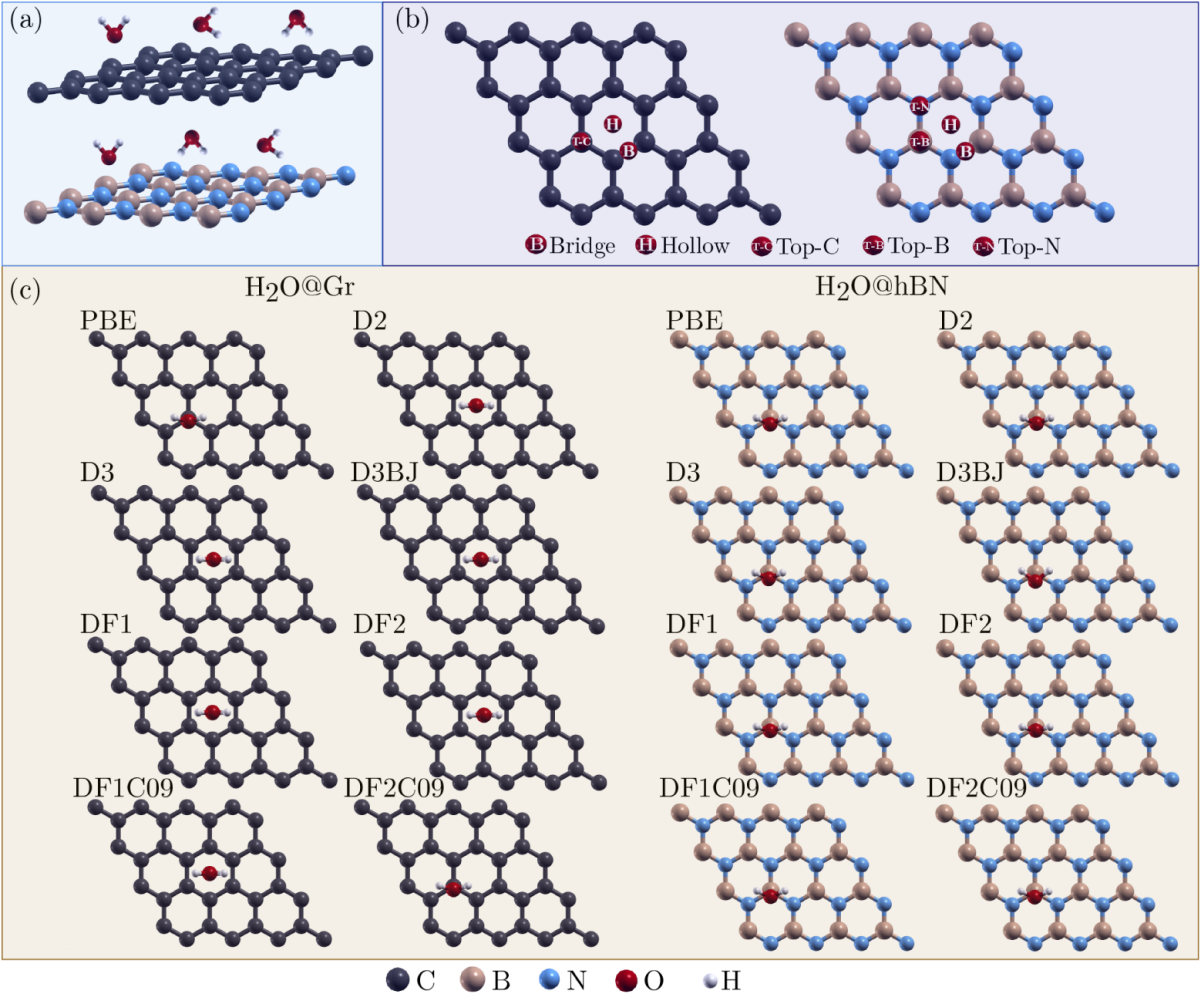
Representative adsorption geometries of a single H_2_O molecule on graphene (Gr) and hexagonal boron nitride (hBN) monolayers. (a) Possible water geometry positions on Gr and hBN. (b) High-symmetry adsorption sites considered for Gr (Bridge, Hollow, and Top-C) and hBN (Bridge, Hollow, Top-B, and Top-N), highlighting the intrinsic chemical asymmetry of the B–N lattice. (c) Optimized adsorption configurations corresponding to the most stable geometries obtained from the PBE + vdW calculations. Top and side views are shown to emphasize the adsorption geometry and the orientation of the water dipole relative to the surface. Carbon, boron, nitrogen, oxygen, and hydrogen atoms are represented by gray, pink, blue, red, and white spheres, respectively.

**Table 1:**
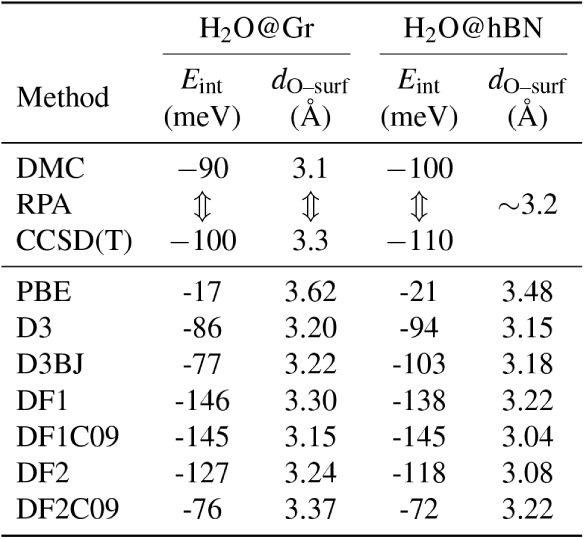
Comparison between PBE + vdW Adsorption Energies and Equilibrium O–Surface Distances for H_2_O Adsorbed on Gr and hBN, Contrasted with Many-Body Reference Benchmarks^a^

a The interaction energies (*E*_int_, eV) for H_2_O adsorbed on Gr and hBN are presented for their most stable adsorption sites (Hollow for Gr and Top-B for hBN). Benchmark adsorption energies and equilibrium distances are taken from DMC, RPA, and CCSD­(T) calculations reported in Refs. 25,26 .

The many-body reference methods such as DMC, RPA, and CCSD­(T) remain the current standard for assessing dispersion-inclusive DFT approaches,^3,25,26^ providing a robust and widely accepted benchmark for weak physisorption systems. Consequently, Table 1 places the present PBE+vdW results in direct correspondence with high-level many-body reference data. For H_2_O@Gr, DMC, RPA, and CCSD­(T) benchmarks consistently report adsorption energies in the range of −90 meV to −100 meV, with equilibrium O–surface distances between 3.1 and 3.3 Å. Among the tested schemes, D3 and D3BJ provide the closest quantitative agreement with the benchmark adsorption energies. DF2 reproduces equilibrium adsorption distances reasonably well, although it moderately overestimates the interaction strength (27–37 meV). DF2C09 yields weaker adsorption energies (similar to D3BJ for H_2_O@Gr case) and slightly overestimates the equilibrium O–surface distance. In contrast, DF1 and DF1C09 overestimate binding by approximately 45–56 meV, a well-documented consequence of the exchange component employed in early vdW-DF formulations.

A closely analogous hierarchy is observed for H_2_O@hBN. While PBE severely underbinds, D3 and D3BJ yield interaction energies in close agreement with lattice-regularized DMC and CCSD­(T) references (−100 meV to −110 meV), whereas DF1-based approaches again display systematic overbinding. DF2 moderately overestimates the interaction strength while slightly underestimating the equilibrium adsorption distances, while DF2C09 underestimates the interaction strength but reproduces equilibrium adsorption distances reasonably well. The consistency of this behavior across two chemically distinct substrates confirms that moderately binding dispersion treatments, whether empirical or nonlocal, provide the most reliable description of water physisorption on two-dimensional materials and offer a sound basis for the subsequent analysis of interfacial diffusion.

#### 3.2.1 Adsorption Sites and Equilibrium Geometries

For graphene, water adsorption at the Top, Bridge, and Hollow sites (Figure 2(b)) yields very similar geometries and interaction energies, reflecting the high symmetry and electronic homogeneity of the π-conjugated carbon lattice. Among these configurations, the Hollow site is consistently identified as the global minimum for all vdW schemes, although the energy differences between sites remain within a few meV, as quantified in Tables S6 and S7 of the Supporting Information. This near-degeneracy indicates an almost isotropic potential-energy surface (PES), a hallmark of weak physisorption on graphene.

In contrast, adsorption on hBN exhibits a clear site selectivity. The Top-B configuration is energetically favored over Top-N, Bridge, and Hollow sites (Figure 2(b)), a consequence of the intrinsic polarity of the B–N bond. The electron-deficient boron atom enhances electrostatic and induction contributions, stabilizing the water dipole when the O atom is positioned above the B site. This asymmetry breaks the site degeneracy observed on Gr and results in a more corrugated PES for hBN.

#### 3.2.2 Interaction Energies and Comparison with Benchmarks

The computed interaction energies span a broad range depending on the vdW treatment, see Figure 3. For H_2_O@Gr, PBE severely underestimates binding (*E*_int_ = −17 meV), as expected for a dispersion-free GGA functional. Inclusion of vdW interactions increases the binding energy to values between −70 and −150 meV. Interaction energies obtained from empirical corrections, such as D3 (−86 meV) and D3BJ (−77 meV), are in excellent agreement with DMC and RPA benchmarks, which report adsorption energies around −90 to −100 meV and equilibrium O–surface distances of ∼3.1–3.3 Å.^25^ Similarly, DF2C09 shows reasonable agreement but slightly underbinds the interaction energy (−76 meV) and adsorption height (3.37 Å) within benchmark uncertainty, consistent with quantum Monte Carlo and RPA reference data.^26,40^ This systematic ordering reflects the trends already identified in Table 1, reinforcing the robustness of the observed hierarchy across substrates.

**Figure 3:**
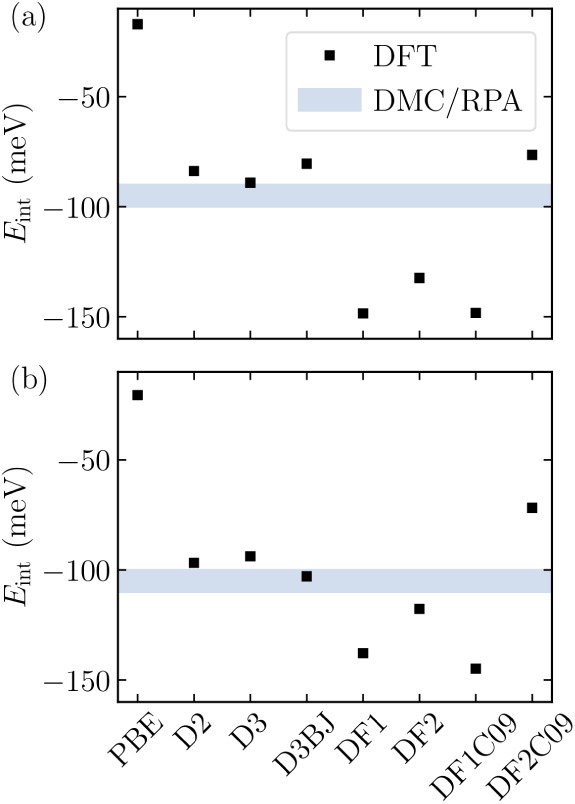
Interaction energies (*E*_int_) for water adsorption on Gr - H_2_O@Gr (a) and hBN - H_2_O@hBN (b) computed using different vdW correction schemes combined with the PBE functional. The shaded horizontal regions indicate the range of adsorption energies obtained from high-level many-body benchmark calculations, including DMC, RPA, and CCSD­(T) methods.^25,26^

On the other hand, DF1 and DF1C09 overestimate the binding of water to graphene, predicting interaction energies close to −150 meV. This behavior is well documented and it is originated from the exchange component employed in early vdW-DF formulations, which is known to exaggerate attractive interactions at intermediate separations.^25,27^ DF2 produces intermediate binding strengths, slightly stronger than benchmarks, but still within an acceptable error margin.

A closely analogous hierarchy is observed for H_2_O@hBN. PBE again underestimates adsorption (−21 meV), while vdW-inclusive schemes predict interaction energies ranging from −70 to −150 meV. D3BJ (−103 meV) and D3 (−94 meV) agree well with lattice-regularized DMC and CCSD­(T) reference values of −100 to −110 meV.^26^ As for graphene, DF1-based approaches overestimate binding by approximately 30–40 meV, whereas DF2C09 slightly underbinds. Overall, D3 and D3BJ provide the closest agreement with benchmark adsorption energetics, while DF2 offers a physically consistent description of adsorption geometries and diffusion behavior despite its moderate overbinding tendency. The trends observed here are also consistent with previous studies involving adsorption of volatile organic compounds and weakly interacting molecules on graphene and related 2D materials, where systematic comparisons between DFT-D methods, nonlocal vdW-DF approaches, high-level reference calculations, and experimental measurements showed that moderately corrected dispersion schemes generally provide the best compromise between adsorption energetics and structural accuracy.^4,25,40^

#### 3.2.3 Charge Redistribution and Nature of the Interaction

Charge-density-difference maps, exemplified in Figure 4 for the Hollow configuration on Gr and the Top-B configuration on hBN, reveal the microscopic origin of the adsorption trends. For both substrates, the interaction is characterized by weak, spatially delocalized charge rearrangement, with electron accumulation around the O atom and depletion in the underlying surface region. This pattern is consistent with the dipole-induced polarization rather than covalent bonding. The effect is stronger on hBN, specifically at the Top-B site, which reflects the enhanced electrostatic response associated with the polar B–N framework. The absence of localized charge sharing or bond formation assures that adsorption remains within the physisorption regime. Projected densities of states for representative adsorption configurations (Figures S17 and S18) confirm the absence of hybridization features close to the Fermi level, consistent with purely physisorptive binding.

**Figure 4:**
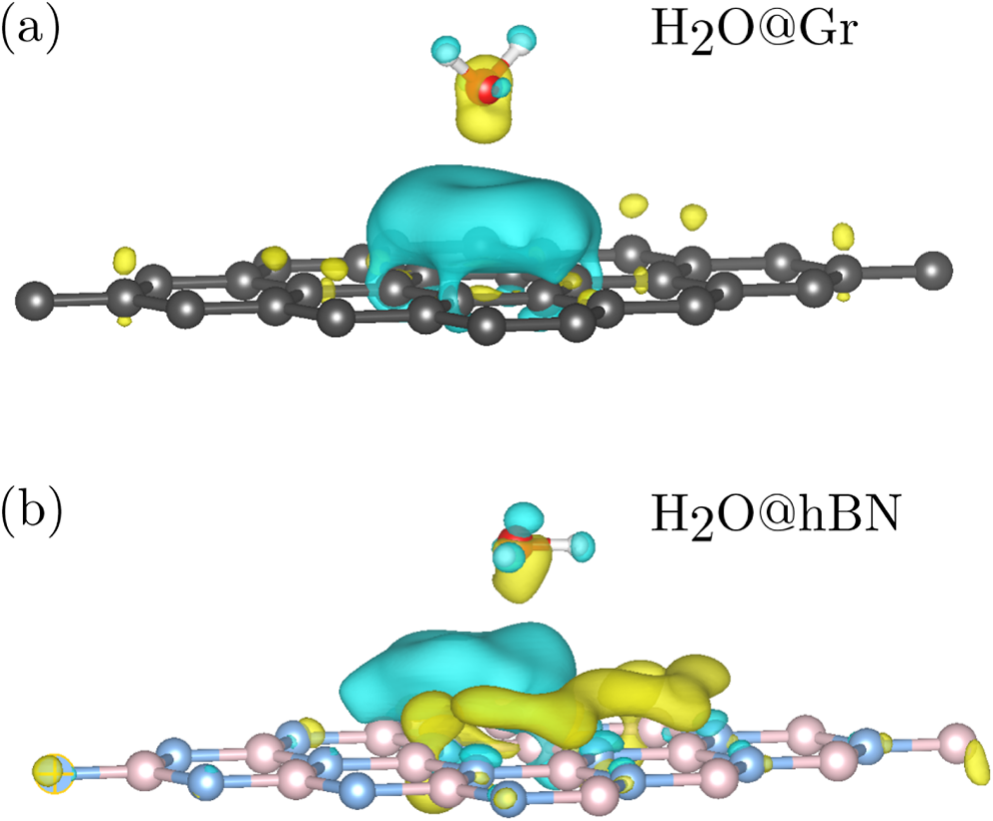
Charge-density difference maps illustrating the electronic redistribution induced by water adsorption on (a) Gr and (b) hBN monolayers. The charge-density difference is defined as $\Delta \rho =\rho_{H_{2}O@\mathrm{surface}}-\rho_{\mathrm{surface}}-\rho_{H_{2}O}$ , where all densities are evaluated at the adsorption geometry. Yellow and cyan isosurfaces represent electron accumulation and depletion, respectively. The maps correspond to the most stable adsorption configurations (Hollow for Gr and Top-B for hBN) obtained using the PBE + D3BJ scheme. The isosurface value is set to ±5 × 10^–4^ e/Bohr^3^.

#### 3.2.4 Implications for Surface Mobility

The small energy differences between adsorption sites on Gr and the modest corrugation on hBN imply low diffusion barriers, a point corroborated by the dynamical analyses discussed in the following subsection. The nearly flat PES on Gr, in particular, explains the facile lateral motion of water molecules observed in AIMD simulations and the sub-0.02 eV migration barriers obtained from CI-NEB calculations. This interpretation is fully consistent with the weak site-to-site energy corrugation (Table S6) and the sub-20 meV diffusion barriers obtained from CI-NEB calculations (Table S8). Such behavior has been reported extensively for weak physisorption processes on atomically flat 2D materials, which provides strong support for the transferability of the present conclusions beyond the specific case of water adsorption.

Overall, the adsorption results establish a coherent physical picture: dispersion interactions dominate binding, electrostatic polarization modulates site preference on hBN, and the subtle balance between exchange and correlation determines the quantitative accuracy of each vdW scheme. The close agreement between the D3 and D3BJ approaches with high-level many-body benchmarks validates their reliability for modeling weakly bound water at 2D material interfaces.

### 3.3 Surface Mobility and Diffusion

Beyond static adsorption energetics, the dynamical behavior of interfacial water provides an additional and sensitive probe of the underlying PES. AIMD simulations at 300 K confirm that isolated H_2_O molecules remain stably physisorbed on both Gr and hBN throughout the simulation window, without desorption or dissociation events. Thermal fluctuations of the total energy remain below 0.1 eV, as depicted in Figure 5(a) and (b) for H_2_O/Gr and H_2_O/hBN, indicating well-equilibrated trajectories and weak molecule–surface coupling. The structural stability of the adsorbed molecule is further supported by the time evolution of internal HOH angles and *d*_O–H_ bond length, reported in the panels (c), (d), (e), and (f), which show slight variations for the H_2_O structure adsorbed and under temperature; O–surface distances in Figure S13 depicts the variations of distances given the changes of adsorbed sites under temperature. These data confirm that thermal fluctuations remain within harmonic-like regimes and do not induce reorientation or incipient desorption. Thus, the lateral motion of adsorbed water is characterized by quasi-free diffusion across the surface. Mean-squared displacement analyses (see Supporting Information) yield diffusion coefficients on the order of 1 × 10^–3^–1 × 10^–2^ cm^2^ s^–1^, depending on the vdW correction. These values are consistent with the shallow corrugation expected for vdW–dominated adsorption and reflect the weak anisotropy of the underlying adsorption landscape.

**Figure 5:**
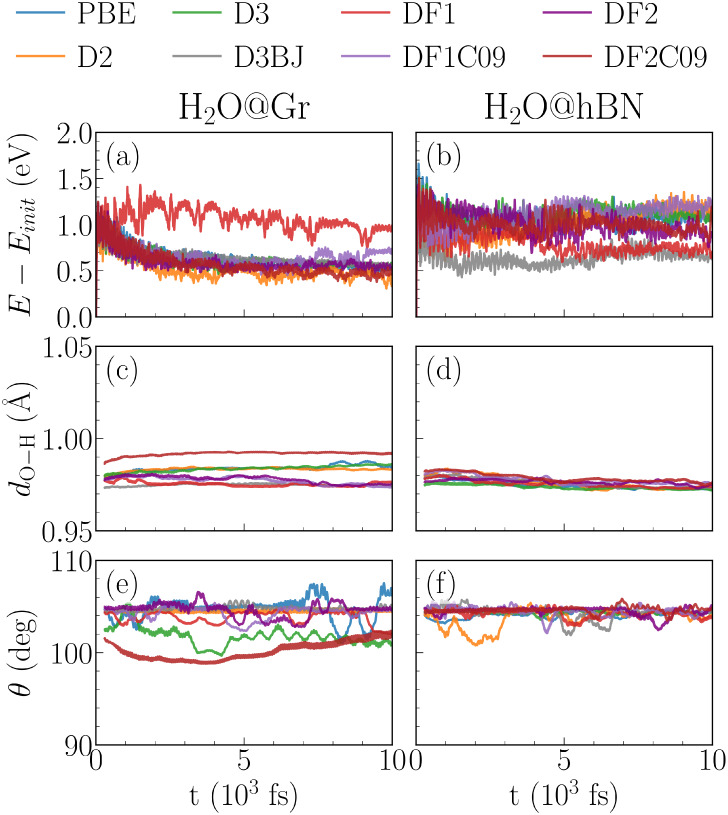
AIMD parameters for *t* = (0 → 10) × 10^3^ fs interval with *E* – *E*_init_ energy, *d*_O–H_ bond length, and HOH θ angle for H_2_O@Gr—(a), (c), and (e)—and H_2_O@hBN—(b), (d), and (f)—for all vdW corrections investigated.

To quantify the microscopic origin of this high mobility, minimum-energy diffusion pathways were computed using the CI-NEB method. Figure 6 displays representative minimum-energy diffusion pathways for water migration between adjacent adsorption sites on Gr and hBN. Across all vdW schemes, one observes that all the associated activation barriers remain below 0.02 eV, comparable to the thermal energy at room temperature (*k*_B_ *T* ≈ 26 meV at 300 K), i.e., confirming that surface diffusion proceeds via a low-barrier hopping mechanism. Moderately binding schemes such as D3, D3BJ, and DF2 yield a balanced description of adsorption energetics and diffusion barriers (5–8 meV), although DF2 systematically predicts stronger interaction energies than the benchmark references.

**Figure 6:**
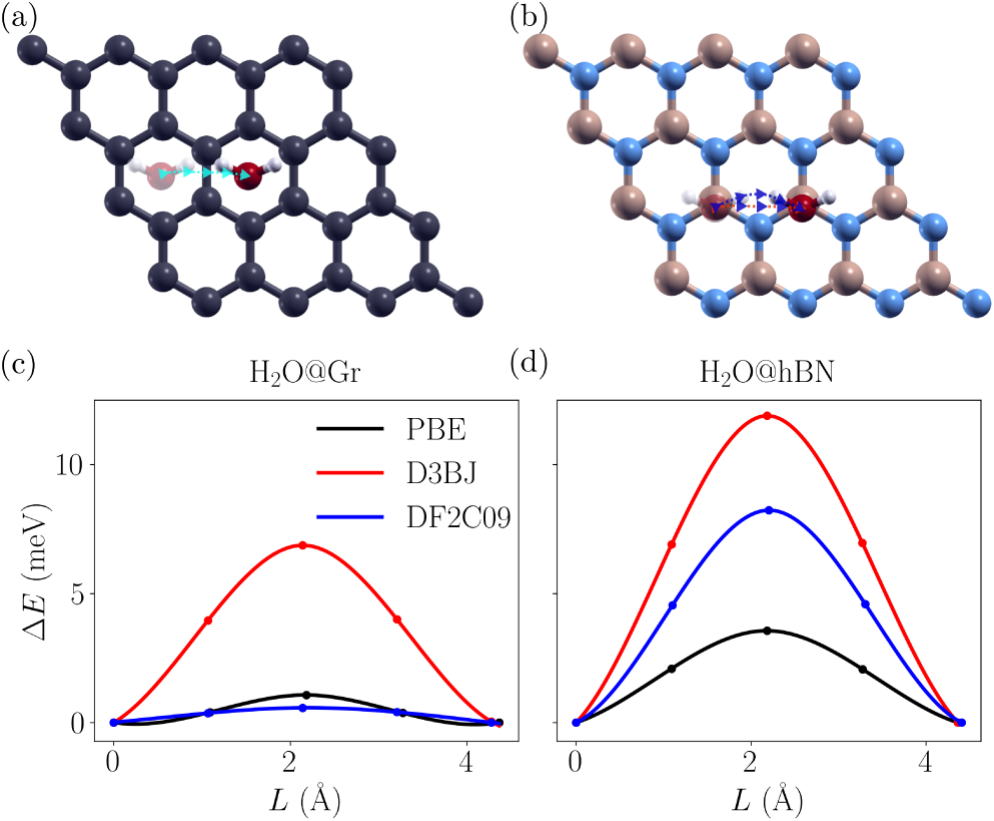
Minimum-energy diffusion pathway for a water molecule migrating between adjacent adsorption sites on Gr (a) and hBN (b) obtained using the climbing-image nudged elastic band (CI-NEB) method. The reaction coordinate corresponds to the lateral displacement of the molecule across the surface. Insets illustrate representative configurations along the diffusion pathway, highlighting the initial adsorption site, the transition-state configuration, and the final state. The energy profile reveals a shallow potential-energy landscape with small diffusion barriers for H_2_O@Gr (c) and H_2_O@hBN (d).

The observed inverse correlation between adsorption strength and mobility is physically transparent: vdW schemes that overestimate binding (e.g., DF1-based functionals) slightly suppress diffusion, whereas methods yielding near-benchmark adsorption energies predict the highest diffusivity. This behavior is fully consistent with DMC and RPA studies, which report an almost isotropic PES for water on atomically flat 2D substrates.^25,26,40^ Overall, these results demonstrate that dispersion interactions not only stabilize adsorption but also critically modulate interfacial dynamics, governing water mobility on Gr and hBN. Detailed diffusion coefficients and activation energies for all vdW schemes are reported in the Supporting Information.

## 4 Conclusions

In this work, we presented a systematic and unified assessment of van der Waals correction schemes for describing water adsorption and diffusion on graphene and hexagonal boron nitride monolayers within density functional theory. By combining structural, energetic, electronic, and dynamical analyses, we clarified how different treatments of long-range correlation impact the accuracy and physical consistency of interfacial water–2D material interactions. Bare PBE severely underestimates adsorption energies on both substrates, reflecting its inability to capture dispersion-driven physisorption. Empirical corrections and nonlocal vdW-DF functionals substantially improve the description, but clear and systematic differences emerge among the tested approaches. Early vdW-DF formulations (DF1 and DF1C09) consistently overbind water, whereas moderate dispersion schemes such as D3 and D3BJ yield adsorption energies and equilibrium distances in the closest agreement with diffusion Monte Carlo and random-phase approximation benchmarks, whereas DF2 reproduces adsorption geometries and diffusion trends reasonably well despite moderately overestimating interaction strengths. Beyond static properties, climbing-image nudged elastic band and *ab initio* molecular dynamics simulations reveal that water diffusion on both graphene and hBN proceeds via a hopping mechanism over a shallow potential-energy surface, with activation barriers below 0.02 eV. Moderately binding vdW schemes preserve high surface mobility, whereas overbinding functionals slightly suppress diffusion. This consistent correlation between adsorption strength and dynamical behavior underscores the importance of a balanced treatment of dispersion, given the significant differences in computational cost among the available vdW approaches. Among the vdW schemes considered, D3 and D3BJ provide the closest overall agreement with high-level many-body benchmark energetics, reproducing adsorption energies and equilibrium distances within the expected accuracy range for both graphene and hBN, while DF2 remains physically consistent in describing equilibrium adsorption geometries and interfacial diffusion. In contrast, while DF2C09 yields interaction energies in the correct magnitude range, it tends to underestimate the binding strength in comparison with the reference values and does not consistently reproduce the benchmark trends for both substrates. In general, these results show that moderately binding dispersion treatments give a balanced and reliable picture of water physisorption on 2D materials, especially when both energetic and dynamical properties are considered. These results reinforce the importance of meticulously selecting vdW treatments based on the balance between interaction strength and transferability, instead of depending on a singular universally optimal approach. The present benchmark-based analysis establishes a robust groundwork for future studies on multilayer water adsorption, confined aqueous phases, and water-mediated processes in vdW heterostructures, catalysis, and nanofluidic applications.

## Supplementary Material



## Notes

Convergence tests for cutoff energy, **k**-point sampling, and supercell size; charge-density difference plots; AIMD temperature and energy profiles; CI-NEB reaction pathways (PDF)
