# Supplementary material for: Assessing van der Waals Corrections in the Description of Water Adsorption and Diffusion on Graphene and Hexagonal Boron Nitride
Source: ACS Omega. 2026 Jun 12;11(25):37771–9. doi: 10.1021/acsomega.6c02762 (PMC13325133; doi:10.1021/acsomega.6c02762)
Supplement: Supplementary file 1 [file ao6c02762_si_001.pdf]

Supporting Information: Assessing van der Waals Corrections in the Description of Water Adsorption and Diffusion on Graphene and Hexagonal Boron Nitride

Tulio Gnoatto Grison,[†] Douglas D. de Vargas,[‡] Celso R. C. Rêgo,[¶] Alexandre Cavalheiro Dias,[§] Mateus Henrique Köhler,[‡] Diego Guedes-Sobrinho,[‖] and Maurício Jeomar Piotrowski[*,†]

†Department of Physics, Federal University of Pelotas, PO Box 354, Pelotas, RS, 96010-900, Brazil

‡Department of Physics, Federal University of Santa Maria, Santa Maria, RS, 97105-900, Brazil

¶Karlsruhe Institute of Technology (KIT), Institute of Nanotechnology, Eggenstein-Leopoldshafen, Germany

§Institute of Physics and International Center of Physics, University of Brasília, Brasília 70919-970, DF, Brazil

‖Chemistry Department, Federal University of Paraná, Curitiba, PR, 81531-980, Brazil

E-mail: mauriciomjp@gmail.com

Contents

I Computational Convergence and Validation S-3

A Plane-Wave Cutoff and k-Point Sampling S-3

B Equilibrium Lattice Parameters S-4

II Electronic Structure of Pristine Graphene and hBN S-7

III Properties of the Isolated Water Molecule S-9

IV Adsorption Energetics and Site Dependence S-10

V Dynamical Properties and Diffusion S-14

VI Projected Density of States S-19

I Computational Convergence and Validation

A Plane-Wave Cutoff and k-Point Sampling

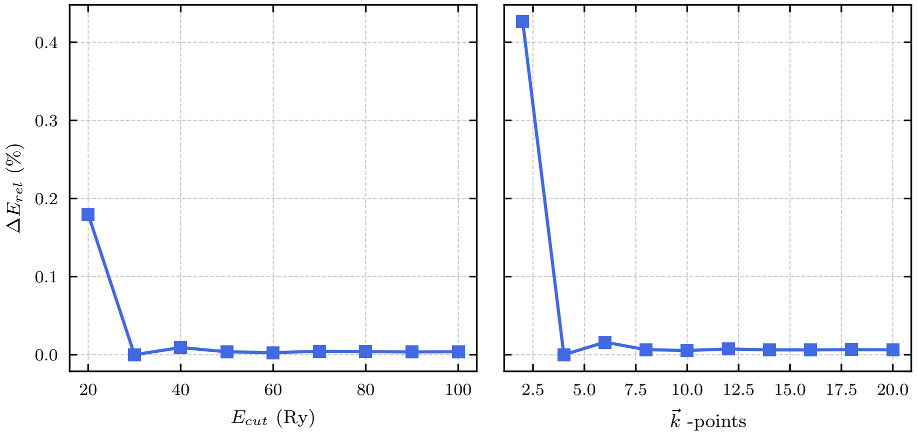

Figure S1: Relative total-energy convergence with respect to plane-wave cutoff energy and Monkhorst–Pack k-point sampling for pristine graphene.

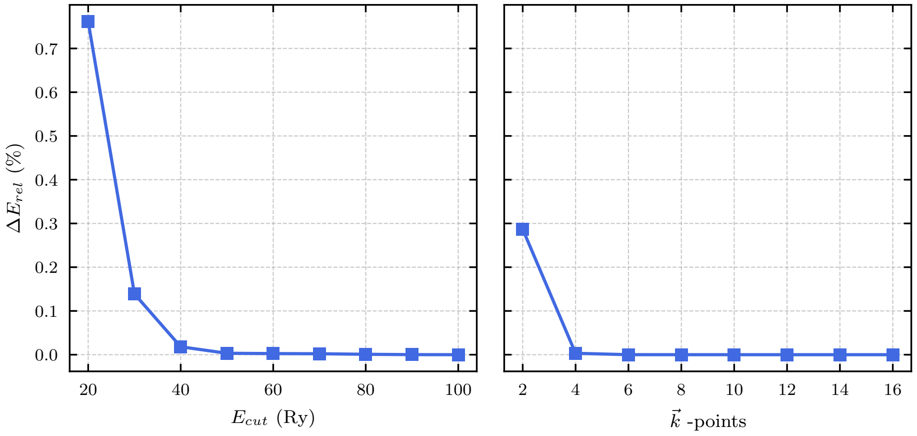

Figure S2: Relative total-energy convergence with respect to plane-wave cutoff energy and Monkhorst–Pack k-point sampling for pristine hBN.

B Equilibrium Lattice Parameters

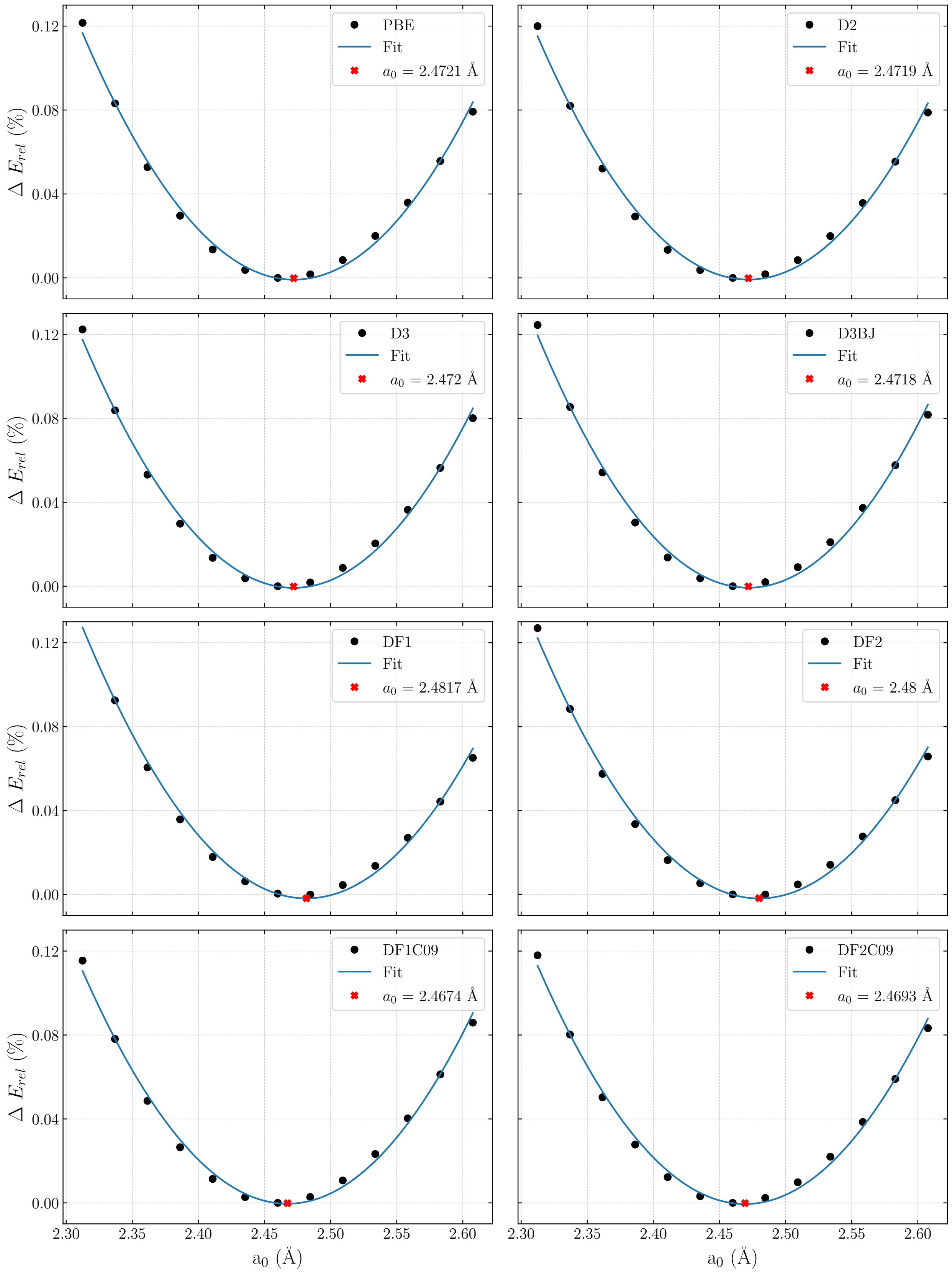

Figure S3: Relative total energy as a function of in-plane lattice parameter for graphene using different vdW schemes.

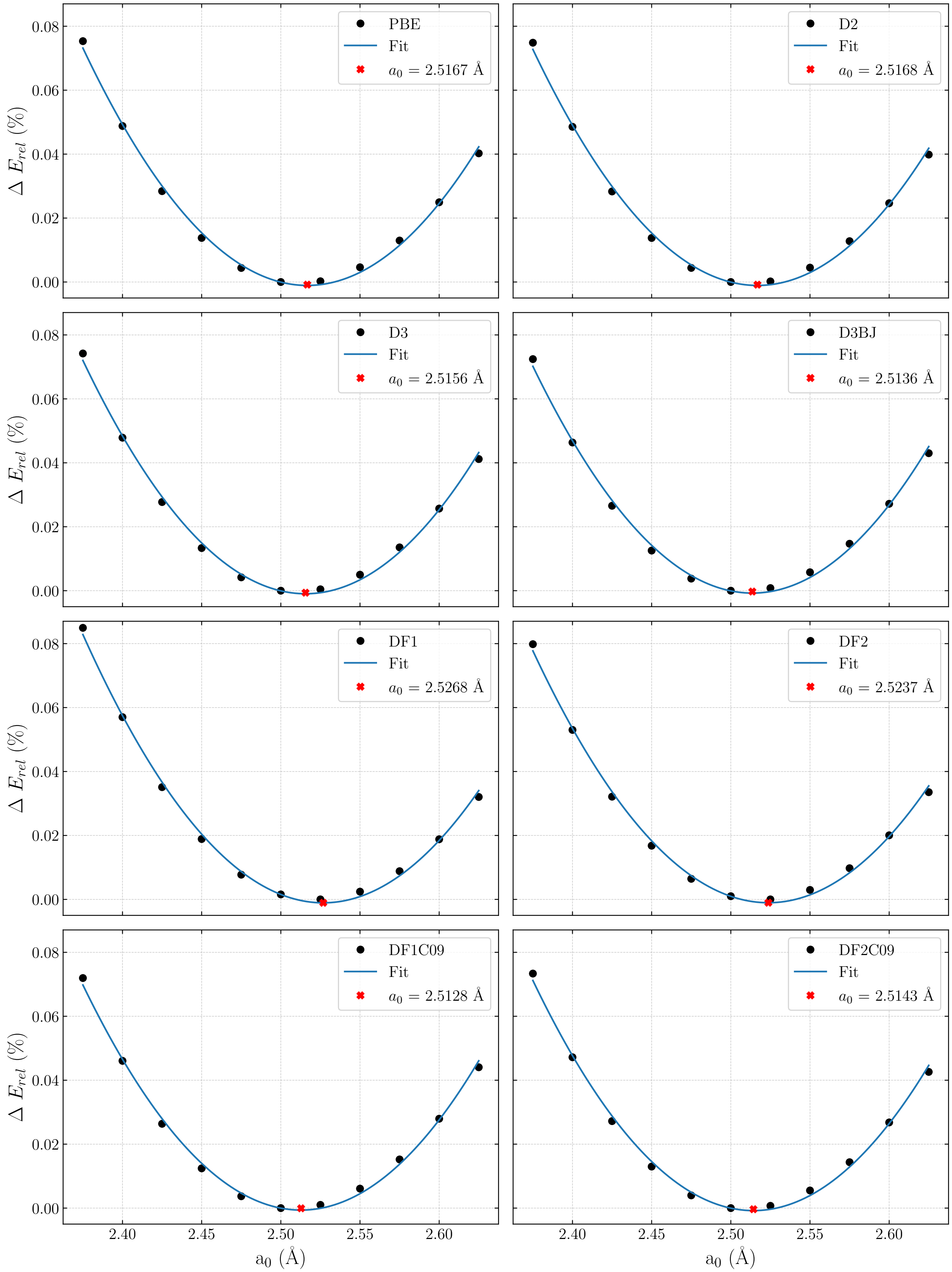

Figure S4: Relative total energy as a function of in-plane lattice parameter for hBN using different vdW schemes.

Table S1: Equilibrium lattice parameters and relative errors with respect to experimental values for Gr and hBN.

vdW	a_{Gr} (Å)	Error (%)	a_{hBN} (Å)	Error (%)
PBE	2.4721	0.41	2.5167	0.55
D2	2.4719	0.40	2.5168	0.55
D3	2.4720	0.41	2.5156	0.50
D3BJ	2.4718	0.40	2.5136	0.42
DF1	2.4817	0.80	2.5268	0.95
DF1C09	2.4674	0.22	2.5128	0.39
DF2	2.4800	0.73	2.5237	0.83
DF2C09	2.4693	0.30	2.5143	0.45

II Electronic Structure of Pristine Graphene and hBN

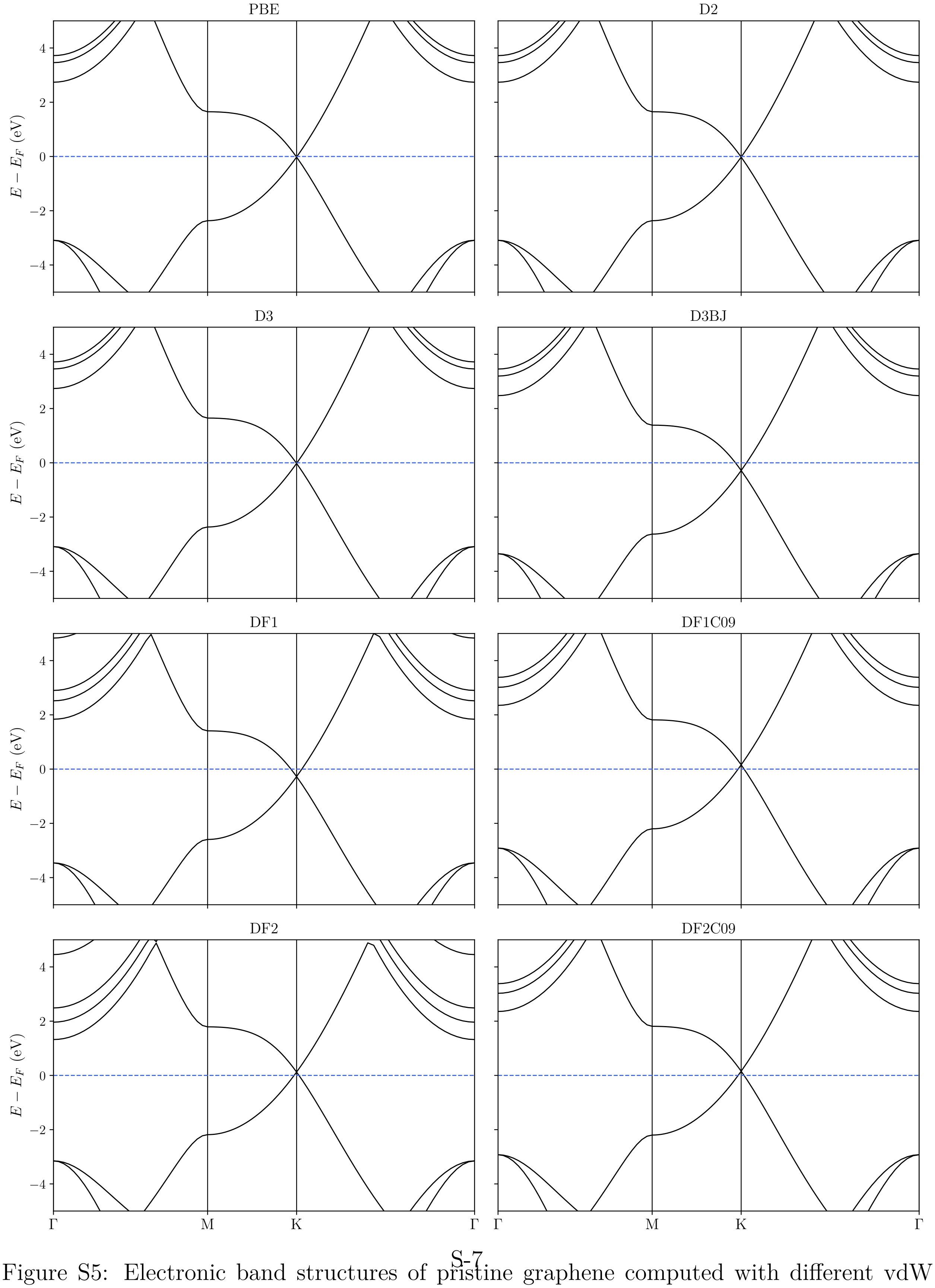

Figure S5: Electronic band structures of pristine graphene computed with different vdW schemes.

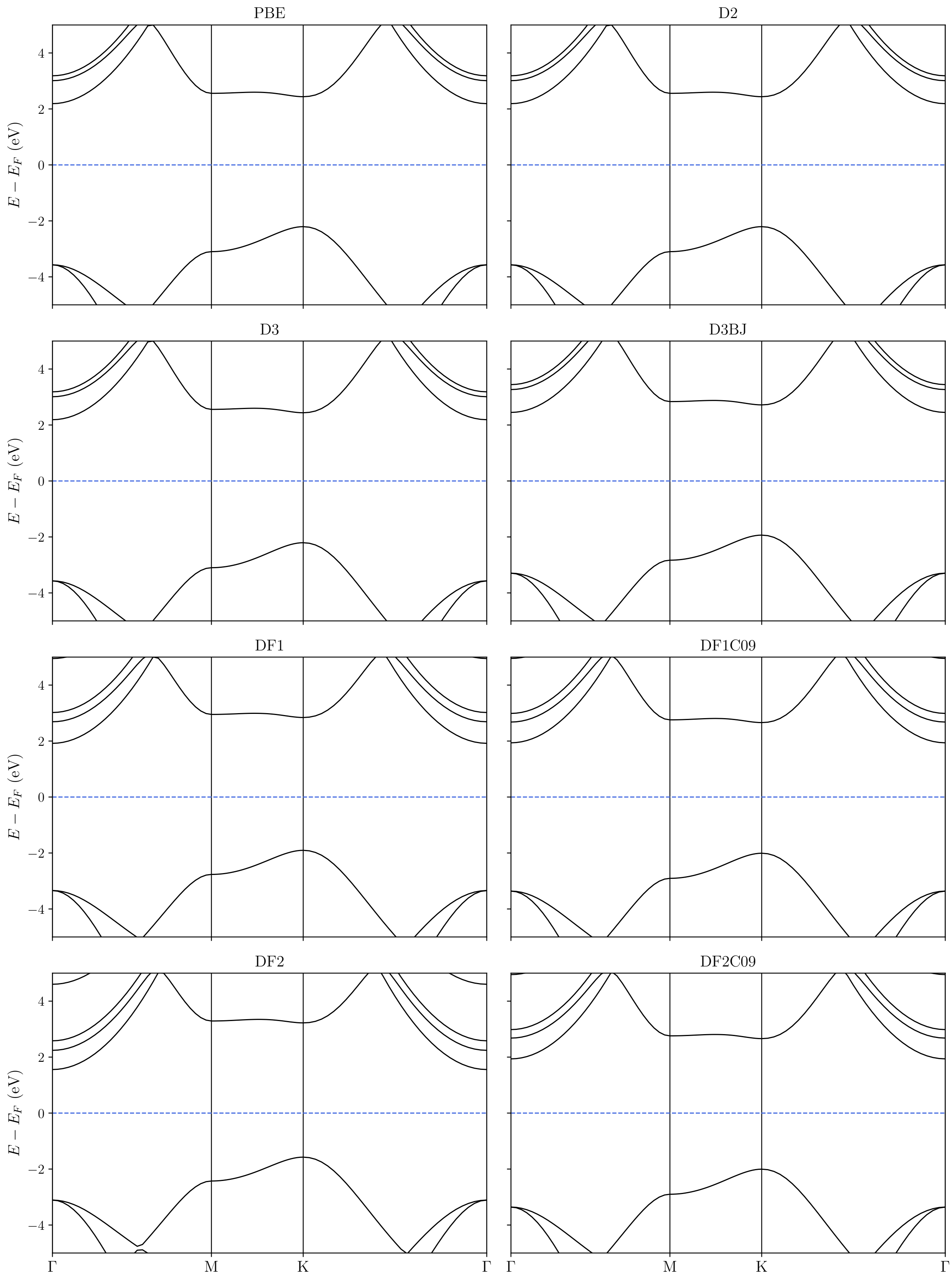

Figure S6: Electronic band structures of pristine hBN computed with different vdW schemes.

Table S2: Indirect K–Γ band gap of hBN obtained with different vdW corrections.

vdW	E_{gap} (eV)
PBE	4.395
D2	4.395
D3	4.395
D3BJ	4.384
DF1	3.823
DF1C09	3.944
DF2	3.130
DF2C09	3.941

III Properties of the Isolated Water Molecule

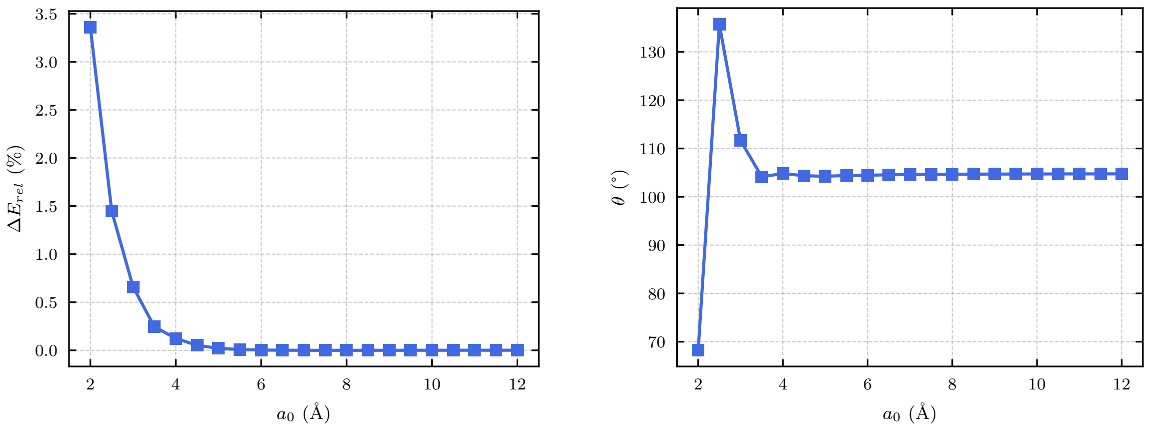

(a) (b)

Figure S7: Convergence of (a) relative total energy and (b) HOH angle of an isolated H_2O molecule as a function of vacuum separation.

Table S3: Structural parameters and binding energy of isolated H_2O obtained with different vdW schemes.

vdW	d_{OH} (Å)	∠HOH (deg)	E_{bind} (eV)
PBE	0.970	104.3	-4.63
D2	0.970	104.4	-4.63
D3	0.970	104.4	-4.63
D3BJ	0.970	104.4	-4.64
DF1	0.970	104.3	-4.40
DF1C09	0.972	104.6	-4.57
DF2	0.970	104.7	-4.38
DF2C09	0.972	104.6	-4.54

IV Adsorption Energetics and Site Dependence

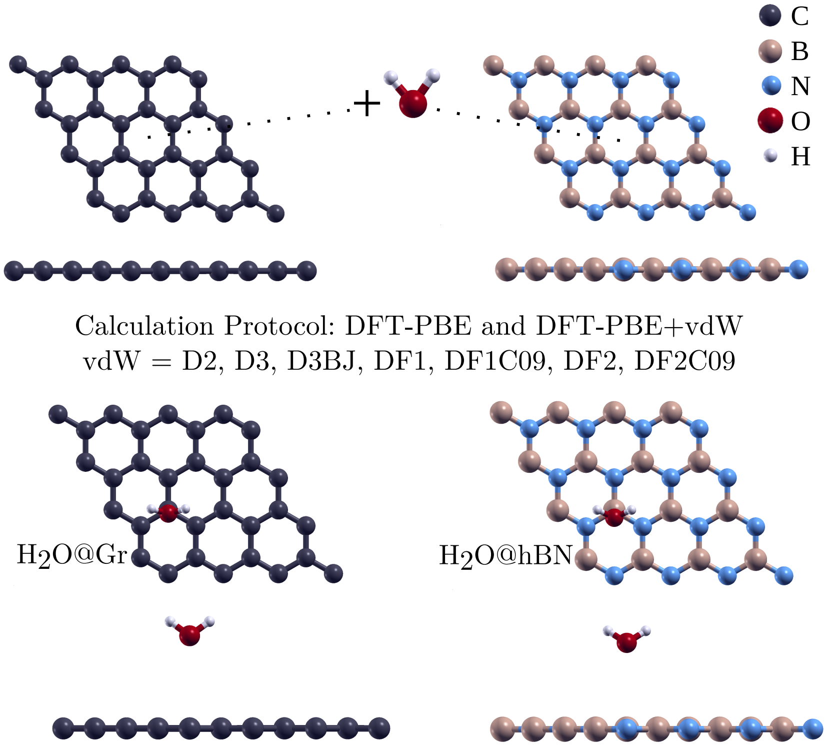

Figure S8: Optimized adsorption geometry of H_2O on graphene and hBN at the most stable sites.

Table S4: Structural parameters and interaction energies for H_2O@Gr at the Top C site.

	PBE	D2	D3	D3BJ	DF1	DF1C09	DF2	DF2C09
$d_{\text{O-H}}$ (Å)	0.97	0.97	0.97	0.97	0.97	0.97	0.97	0.97
θ (deg)	104.7	104.7	104.7	104.7	104.5	104.8	104.9	104.9
$d_{\text{O-surf}}$ (Å)	3.62	3.17	3.20	3.22	3.30	3.15	3.24	3.37
E_{int} (eV)	-17	-79	-86	-77	-146	-145	-127	-76

Table S5: Structural parameters and interaction energies for H_2O@hBN at the Top B site.

	PBE	D2	D3	D3BJ	DF1	DF1C09	DF2	DF2C09
$d_{\text{O-H}}$ (Å)	0.97	0.97	0.97	0.97	0.97	0.97	0.97	0.97
θ (deg)	104.3	104.7	104.6	104.3	104.4	104.8	104.9	104.7
$d_{\text{O-surf}}$ (Å)	3.48	3.05	3.15	3.18	3.22	3.04	3.08	3.22
E_{int} (meV)	-21	-10	-94	-103	-138	-145	-118	-72

Table S6: Interaction energies (E_{int}, eV) of H_2O@Gr for different adsorption sites and vdW schemes.

vdW scheme	Site	E_{int} (meV)
PBE	Top C	-17.1
	Bridge	-14.9
	Hollow	-15.5
D2	Top C	-78.7
	Bridge	-76.3
	Hollow	-83.8
D3	Top C	-85.7
	Bridge	-83.1
	Hollow	-89.1
D3BJ	Top C	-76.9
	Bridge	-74.2
	Hollow	-80.4
DF1	Top C	-146.3
	Bridge	-144.6
	Hollow	-148.4
DF2	Top C	-127.1
	Bridge	-126.1
	Hollow	-132.4
DF1C09	Top C	-145.1
	Bridge	-142.5
	Hollow	-148.2
DF2C09	Top C	-76.5
	Bridge	-74.0
	Hollow	-74.3

Table S7: Interaction energies (E_{int}, eV) of H_2O@hBN for different adsorption sites and vdW schemes.

vdW scheme	Site	E_{int} (meV)
PBE	Top B	-20.6
	Top N	-9.8
	Bridge	-12.3
	Hollow	-17.7
D2	Top B	-96.8
	Top N	-74.1
	Bridge	-82.0
	Hollow	-92.3
D3	Top B	-93.8
	Top N	-72.6
	Bridge	-79.6
	Hollow	-88.0
D3BJ	Top B	-102.9
	Top N	-64.7
	Bridge	-70.7
	Hollow	-79.5
DF1	Top B	-137.8
	Top N	-127.6
	Bridge	-131.1
	Hollow	-134.9
DF2	Top B	-117.7
	Top N	-102.4
	Bridge	-108.2
	Hollow	-114.6
DF1C09	Top B	-144.9
	Top N	-123.0
	Bridge	-131.2
	Hollow	-138.3
DF2C09	Top B	-71.8
	Top N	-57.4
	Bridge	-61.3
	Hollow	-67.6

V Dynamical Properties and Diffusion

Table S8: Diffusion coefficients and activation energies for H_2O@Gr obtained from AIMD and CI-NEB calculations.

vdW scheme	D (cm^2/s)	E_{act} (meV)	Path length (Å)
PBE	0.007	8	4.28
D2	0.035	8	4.28
D3	0.035	7	4.28
D3BJ	0.085	7	4.28
DF1	0.020	4	4.30
DF2	0.069	6	4.30
DF1C09	0.082	7	4.27
DF2C09	0.011	7	4.27

Table S9: Diffusion coefficients and activation energies for H_2O@hBN obtained from AIMD and CI-NEB calculations.

vdW scheme	D (cm^2/s)	E_{act} (meV)	Path length (Å)
PBE	0.019	5	4.51
D2	0.008	12	4.35
D3	0.021	14	4.34
D3BJ	0.060	12	4.37
DF1	0.062	10	5.62
DF2	0.014	16	5.84
DF1C09	0.007	20	5.38
DF2C09	0.019	11	4.79

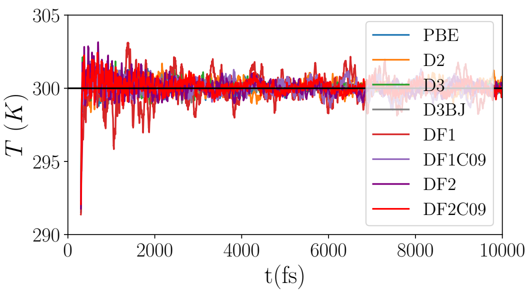

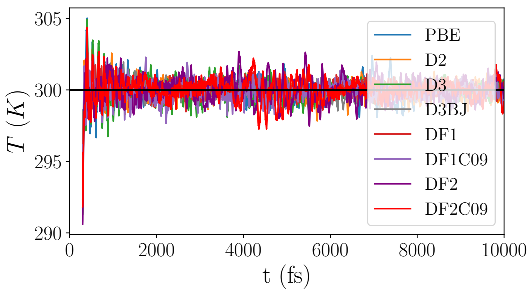

Figure S9: Temperature evolution during AIMD simulations at 300 K for H_2O adsorbed on graphene (left) and hBN (right).

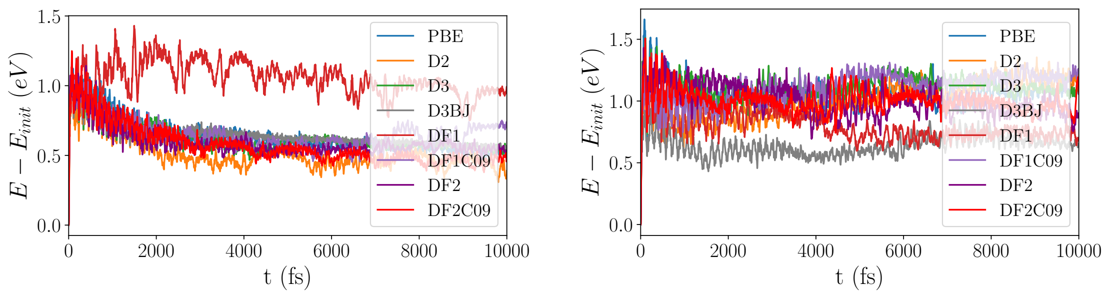

Figure S10: Total-energy fluctuations during AIMD simulations of H_2O on graphene (left) and hBN (right).

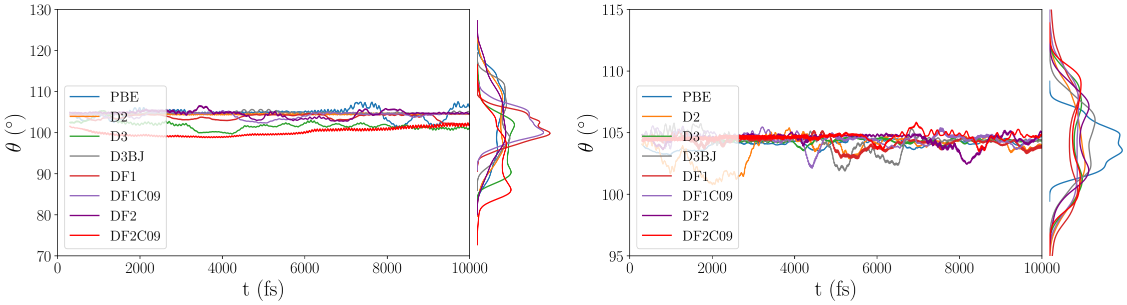

Figure S11: Time evolution of the HOH angle of the adsorbed H_2O molecule during AIMD simulations at 300 K on graphene (left) and hBN (right).

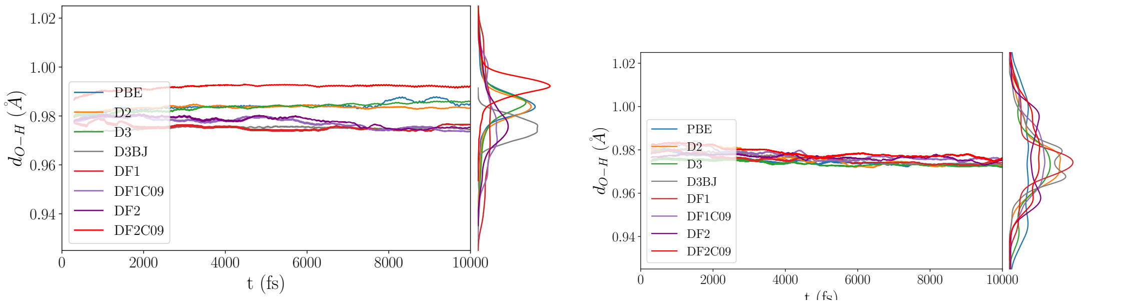

Figure S12: Time evolution of the O–H bond length of the adsorbed H_2O molecule during AIMD simulations at 300 K on graphene (left) and hBN (right).

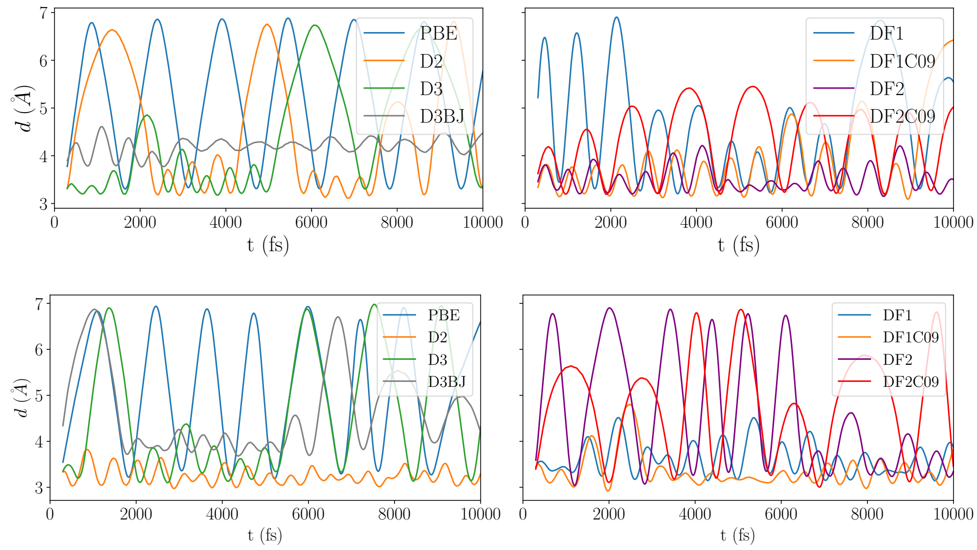

Figure S13: Instantaneous oxygen–surface distance during AIMD simulations of H_2O adsorbed on graphene (top) and hBN (bottom) at 300 K.

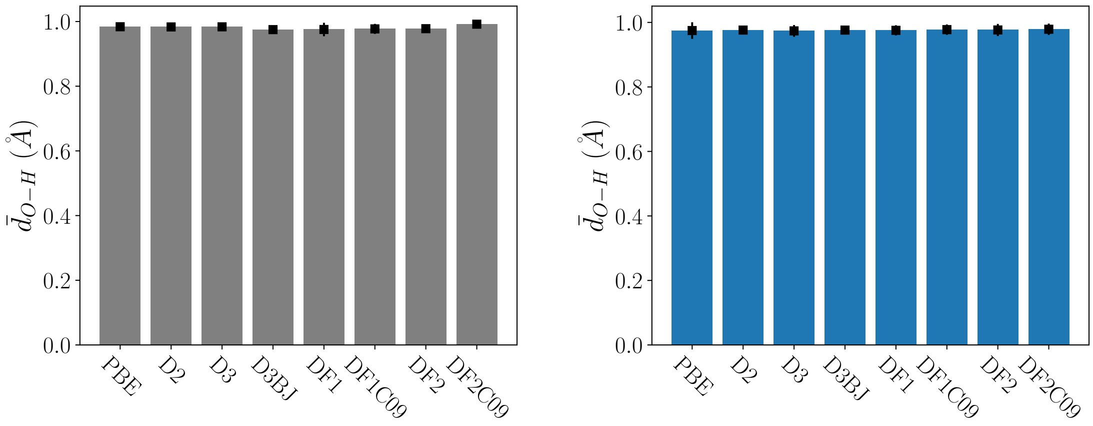

Figure S14: Time-averaged O–H bond length of the H_2O molecule during AIMD simulations on graphene (left) and hBN (right).

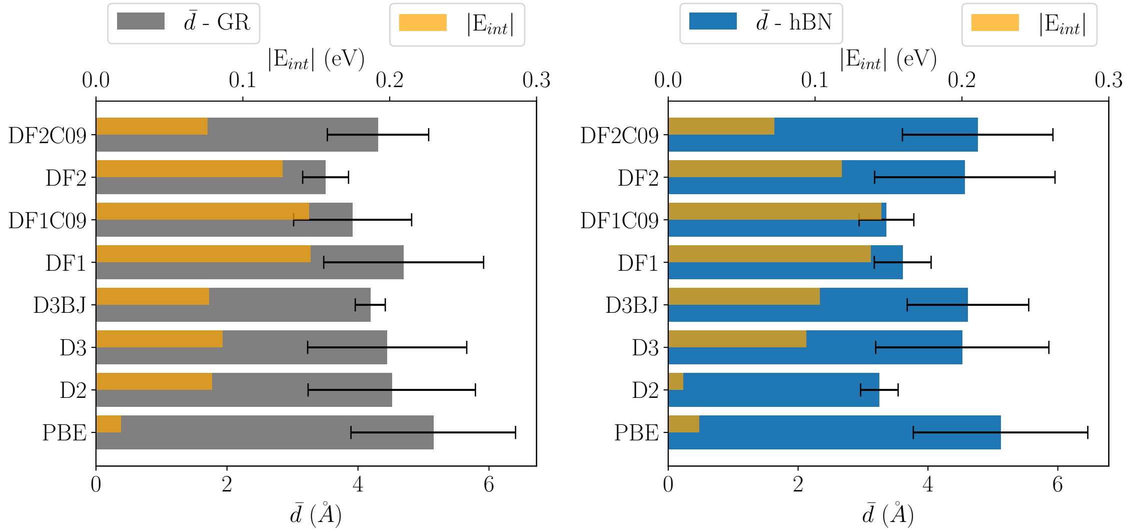

Figure S15: Time-averaged oxygen–surface distance during AIMD simulations of H_2O adsorbed on graphene (left) and hBN (right).

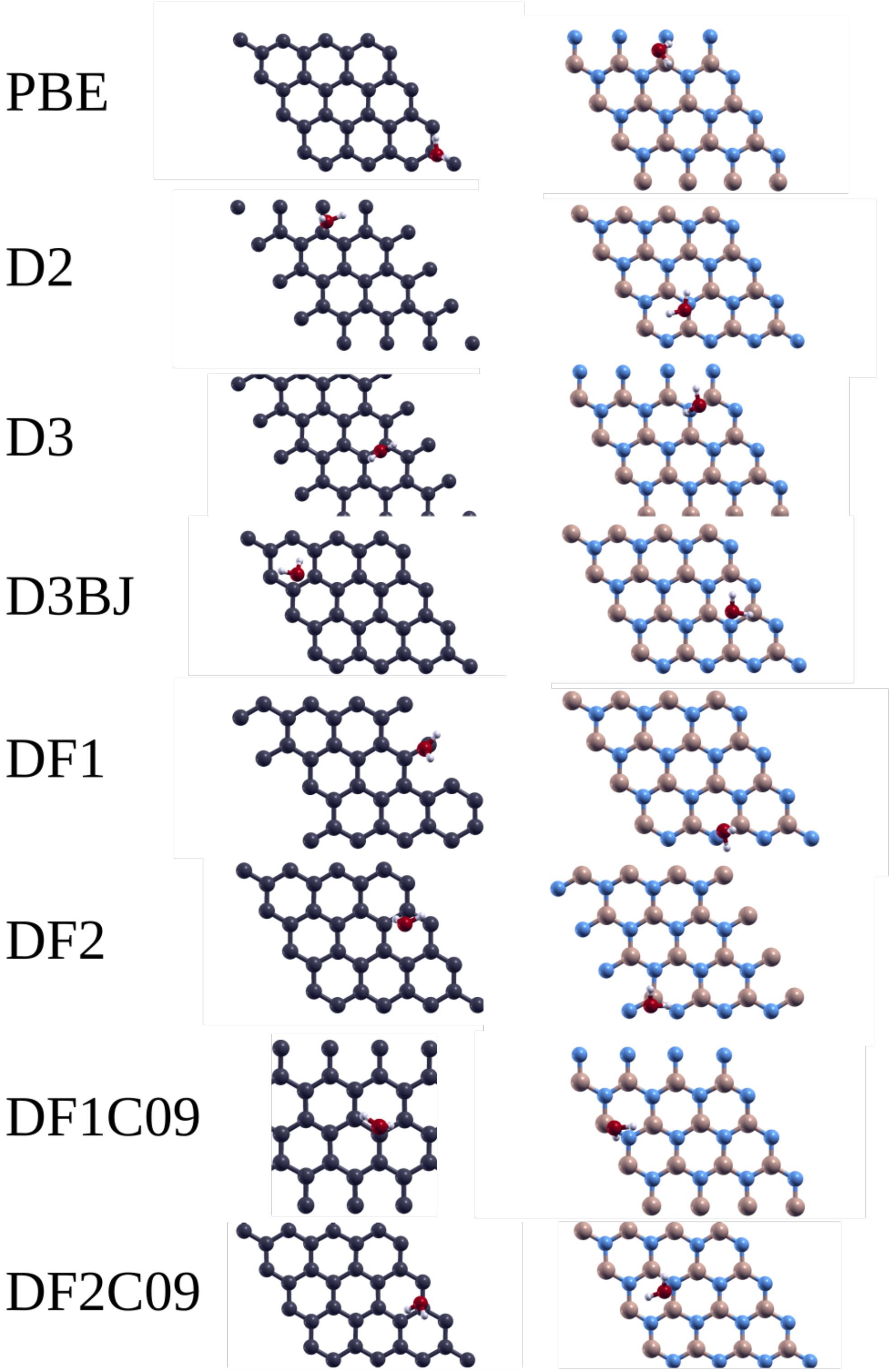

Figure S16: Final atomic configurations of H_2O adsorbed on graphene and hBN after AIMD simulations at 300 K for representative vdW schemes.

VI Projected Density of States

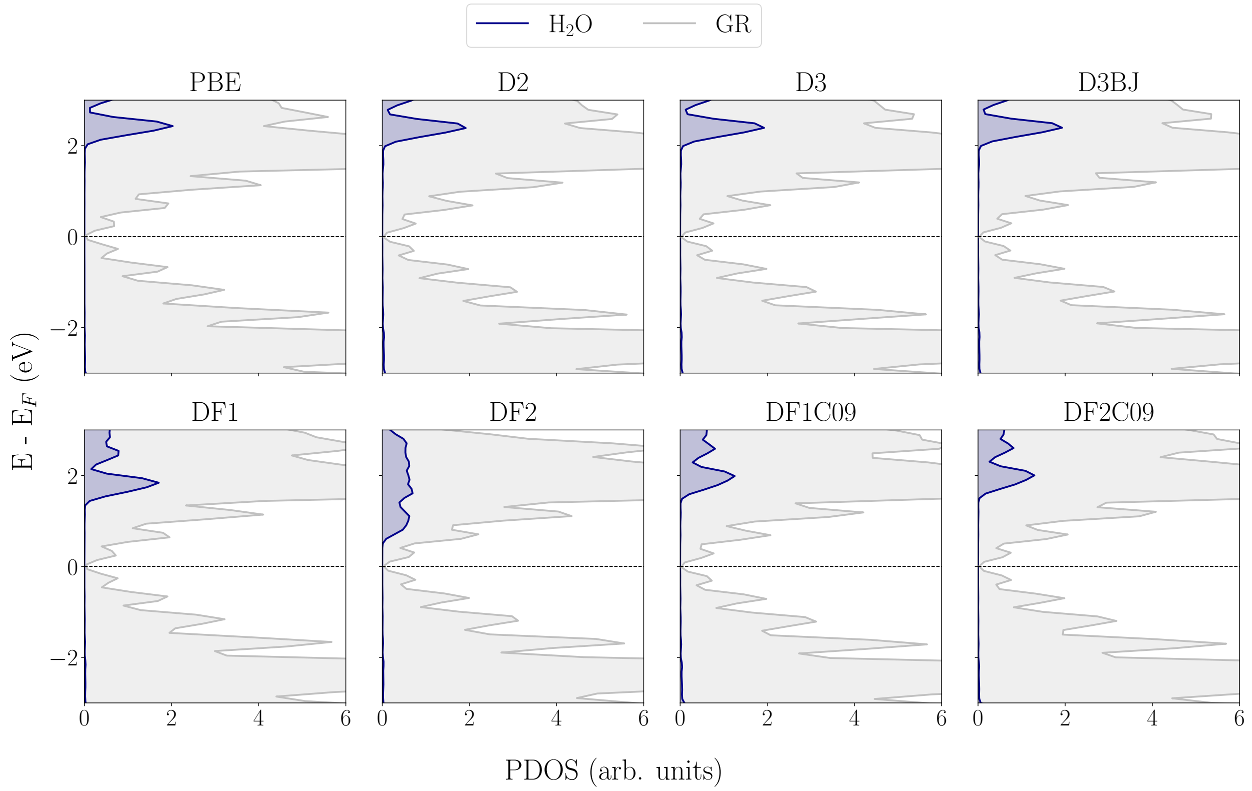

Figure S17: Projected density of states of H_2O@Gr for representative vdW schemes.

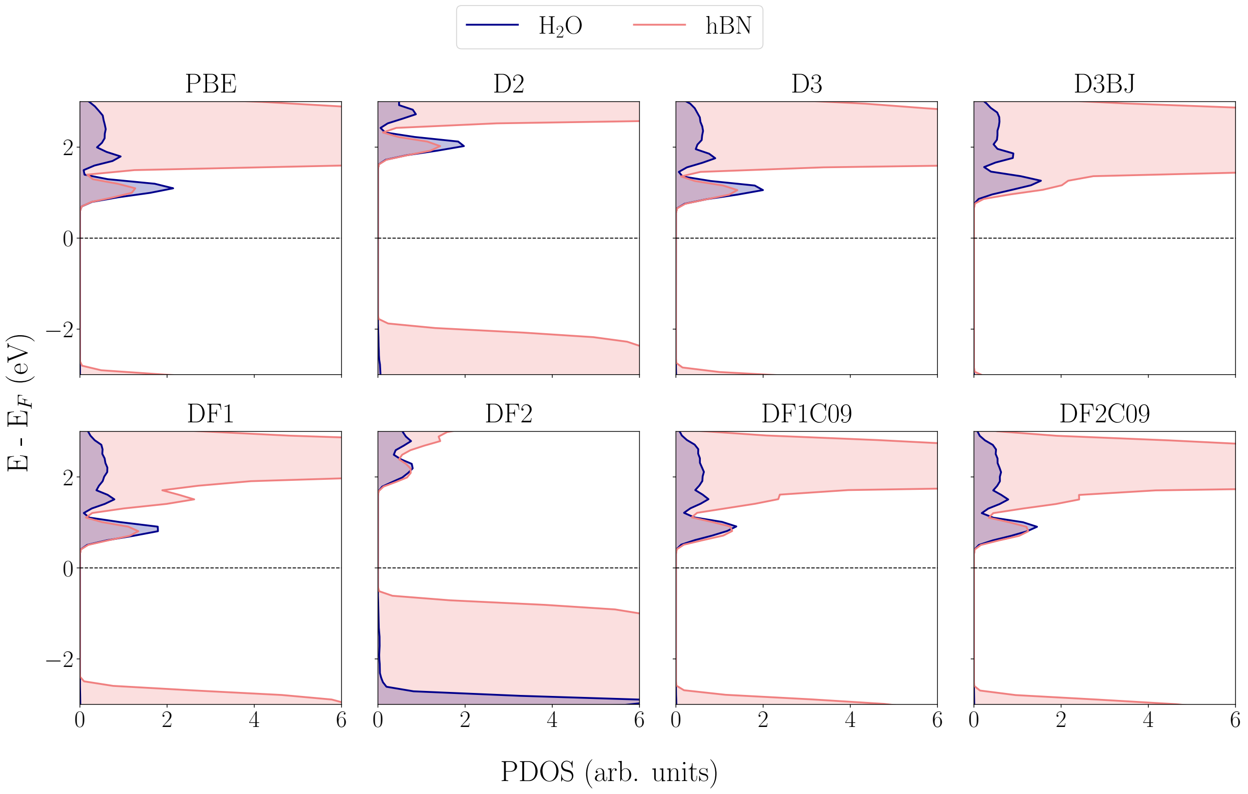

Figure S18: Projected density of states of H_2O@hBN for representative vdW schemes.
